# Scientific Items

**Published:** 1883-11-20

**Authors:** 


### SCIENTIFIC ITEMS.

**Gelatine Test for Organisms in Water.**—Dr. Angus Smith, of Manchester, has recently brought forward a new test for the detection of organisms in water. It consists in rendering the water thick by dissolving gelatine in it. If the water is pure, the gelatine cylinder remains long unaltered; but, if it is impure from the presence of organisms, the gelatine round these becomes liquefied and globular, the organisms remaining solid at the bottom of the spheres. Dr. Angus Smith has prepared photographs of test-tubes of water which had been thickened by a solution of the purest fish gelatine, and then exposed to the action of light. When the water was pure, it remained translucent; but when bad, bubbles were rapidly formed, and the bacteria which appeared to be in the water began to act on the gelatine, breaking it up and rendering it soluble. A rapid movement of gas was observable. When the bubbles or balls appeared to be spherical, they indicated aggregations of bacteria. This change took place quickly, almost in twenty-four hours But the test was only applicable where infusoria or fungi were present. For instance, peaty water free from animalcules or bacteria would stand without breaking up the gelatine. To change the gelatine, organisms must be present. Organic matter that is not putrescent or infective will not do it.—*Pop. Sci. News*.

**Discovery of Prehistoric Men.**—Recently, while a new gallery was being pierced in a coal-mine at Bully-Grevay, in the French Department of Pas-de-Calais (*Lancet*), a cavern was opened where the fossil remains of five human beings were found, a man seven feet tall, two women six and six-and-a-half feet, and two children about four feet Fragments of arms and utensils of stone and petrified wood, with remains of mammals and fish were also brought to light. A second chamber enclosed remains of eleven human bodies of large size, several animals, precious stones, etc. The walls exhibited drawings of men fighting with gigantic animals. The petrified bodies were brought to the surface and will be examined by experts from the Academie des Sciences and the British Museum.—*Maryland Medical Journal.*

**Liquid Oxygen and Nitrogen.**—According to the latest researches oxygen when cooled to 137° C. (213° F.) liquifies to a colorless transparent liquid at the very moderate pressure of 23 atmospheres or thereabouts. Nitrogen at the same temperature, when the pressure is cautiously allowed to fall to a point not lower than 50 atmospheres, yields a colorless liquid with distinct meniscus. Ozone under quite moderate limits of pressure and temperature, is a liquid of intensely blue color which gives a vapor which can only be compared in color with the brightest blue sky. Pure alcohol is a white solid at about 130° C. (202° F.). At a very slightly higher temperature it is luicous like oil.—*Lancet.*

**How to Mix Paints.**—The following table, the source of which we are unable to trace at this moment, though we can vouch for its trustworthiness, will be found serviceable, especially for *amateurs*, as showing how simple pigments are to be mixed for producing compound colors:

Buff—Mix white, yellow ochre and red.
Chestnut—Red, black and yellow.
Chocolate—Raw umber, red and black.
Claret—Red, umber and black.
Copper—Red, yellow and black.
Dove—White, vermilion, blue and yellow.
Drab—White, yellow ochre, red and black.
Fawn—White, yellow and red.
Flesh—White, yellow ochre and vermilion.
Freestone—Red, black, yellow ochre and white.
French Gray—White, Prussian blue and lake.
Gray—White lead and black.
Gold—White, stone ochre and red.
Green Bronze—Chrome green, black and yellow.
Green Pea—White and chrome green.
Lemon—White and chrome yellow.
Limestone—White, yellow ochre, black and red.
Olive—Yellow, blue, black and white.
Orange—Yellow and red.
Peach—White and vermilion.
Pearl—White, black and blue.
Pink—White, vermilion and lake.
Purple—Violet, with more red and white.
Rose—White and madder lake.
Sandstone—White, yellow ochre, black and red.
Snuff—Yellow and Vandyke brown.
Violet—Red, blue and white.

In the combinations of colors required to produce a desired tint, the first-named color is always the principal ingredient, and the others follow in the order of their importance. Thus, in mixing a limestone tint, white is the principal ingredient, and red the color of which the least is needed. The exact proportions of each color must be determined by experiment with a small quantity. It is best to have the principal ingredient thick, and add to it the other paints thinner.—*Popular Science News*,

**Strength of Insects.**—At a meeting of the Maryland Academy of Sciences, Dr. Theobald showed a species of beetle, and gave the following figures: weight of beetle, 2 grains; weight moved by it, 5½ ounces or 2,640 grains, or 1,320 times the weight of the beetle. A man weighing 150 pounds, endowed with the strength of this insect, should therefore be able to move 198,000 pounds, or nearly 100 tons.

**A six-pound pickerel**, caught near Shelby, Iowa, had attached to it a complete set of fishing tackle, except the pole.

